# Machine learning bandgaps of double perovskites

**DOI:** 10.1038/srep19375

**Published:** 2016-01-19

**Authors:** G. Pilania, A. Mannodi-Kanakkithodi, B. P. Uberuaga, R. Ramprasad, J. E. Gubernatis, T. Lookman

**Affiliations:** 1 Materials Science and Technology Division, Los Alamos National Laboratory, Los Alamos 87545, NM, USA; 2 Department of Materials Science & Engineering and Institute of Materials Science, University of Connecticut, Storrs, 06269 CT USA; 3 Theoretical Division, Los Alamos National Laboratory, Los Alamos 87545, NM, USA

## Abstract

The ability to make rapid and accurate predictions on bandgaps of double perovskites is of much practical interest for a range of applications. While quantum mechanical computations for high-fidelity bandgaps are enormously computation-time intensive and thus impractical in high throughput studies, informatics-based statistical learning approaches can be a promising alternative. Here we demonstrate a systematic feature-engineering approach and a robust learning framework for efficient and accurate predictions of electronic bandgaps of double perovskites. After evaluating a set of more than 1.2 million features, we identify lowest occupied Kohn-Sham levels and elemental electronegativities of the constituent atomic species as the most crucial and relevant predictors. The developed models are validated and tested using the best practices of data science and further analyzed to rationalize their prediction performance.

In the recent past, high throughput explorations of enormous chemical spaces have significantly aided the rational materials design and discovery process^1^,^2^,^3^,^4^. Massive open-access databases of computed/predicted materials properties (including electronic structure, thermodynamic and structural properties) are now available^5^,^6^,^7^. Materials scientists are currently looking at efficient ways to extract knowledge and mine trends out of materials big-data^8^. As a result, well-established statistical techniques of machine learning (ML) are gradually making inroads into materials science^9^. These methods of data-science and information theory have already met phenomenal success in the fields of cheminformatics^10^, game theory^11^, pattern recognition^12^, artificial intelligence^13^, event forecasting^14^ *etc.* and are now being customized for materials informatics to help identify next generation materials breakthroughs and process optimizations^15^,^16^.

Given past knowledge—in terms of high quality data on a given property of interest for a limited set of material candidates within a well defined chemical space—informatics based statistical learning approaches lead to efficient pathways to make high-fidelity predictions on new compounds within the target chemical space. Some recent examples of materials’ property predictions using informatics include predictions of molecular^17^,^18^ and periodic systems’ properties^19^,^20^,^21^,^22^, transition states^23^, potentials^24^,^25^, structure classifications^26^,^27^,^28^, dielectric properties^2^, self-consistent solutions for quantum mechanics^29^ and predictions of bandgaps^30^,^31^.

In this contribution, we aim to build a validated statistical learning model for a specific class of complex oxides, namely the double perovskites. The double perovskite structure, shown in Fig. 1a, is represented by the chemical formula AA’BB’O_6_; where A and A’ cations are generally of larger radii and have a 12-fold coordination, while the relatively smaller B and B’ metal ions occupy six-fold coordinated positions in oxygen octahedra. The A-site ions typically have +1, +2 or +3 nominal charge states, while the charge state of the B-site cations is governed by the overall charge neutrality of the system. We consider the double perovskite oxides as a material-class of interest owing to both the chemical flexibility made available by the perovskite framework in accommodating a broad spectrum of atomic substitutions, and the vastness of compositional and configurational space spanned by the double perovskites^32^.

The ability to rapidly and accurately predict bandgaps of double perovskites is of much interest for a range of applications that require materials with pre-specified constraints on bandgaps, for instance, scintillation^33^, photovoltaics^34^ and catalysis^35^, to name a few. Local and semi-local functionals used within density functional theory—the current workhorse for electronic structure computations—have a well known deficiency of severely underestimating the bandgaps. More advanced methods such as the GW approach^36^ and hybrid functionals^37^ are enormously computation-time intensive and are thus impractical in high throughput studies aimed at screening promising candidates with targeted bandgaps. This is one of the most important reasons for seeking to develop a statistical (or machine) learning model where one can use easily accessible attributes (also referred to as *features*) of a material to directly predict its bandgap, in an efficient yet accurate manner. Our primary goal is to develop a validated and predictive model that establishes a mathematical relationship (or *mapping*) between the bandgap of material *i* residing in the predefined chemical space, and an Ω-dimensional (Ω−D) feature vector f_*i*_ of the material *i*. Here, the Ω−D vector f_*i*_ (also referred to as a *descriptor*) is composed of Ω different features and uniquely describes the material *i*. It is also desirable to have a model which is both simple (*i.e.*, with sufficiently small Ω) and reasonably accurate. Here, we report a feature-selection (*i.e.*, how to find an optimal Ω−D feature vector) and learning framework (*i.e.*, how to establish the mapping between the bandgaps and feature vectors) for efficient and accurate predictions of electronic bandgaps of double perovskites.

## Results

**Figure 1:**
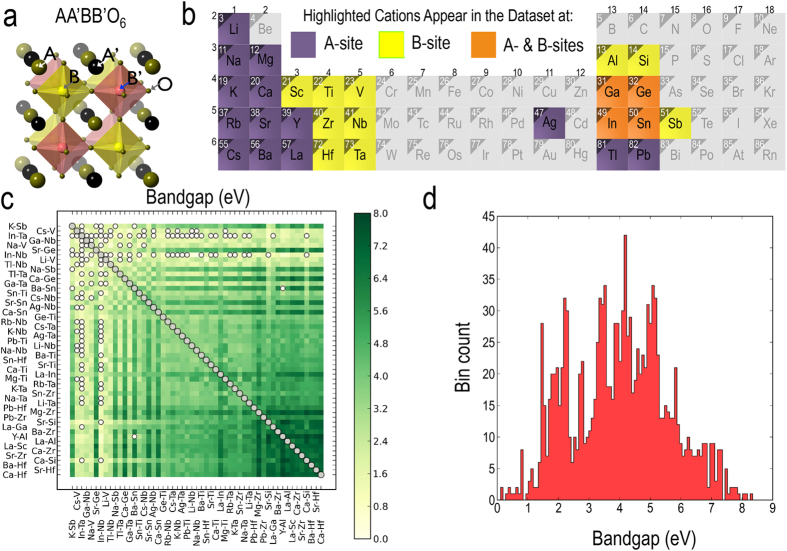
(**a**) Double perovskite crystal structure with rocksalt ordering of both A- and B-site cations. Oxygen octahedral coordination around the B-site cations is explicitly shown. (**b**) Chemical space of the double perovskite oxides explored in the present study. Cations appearing at the A-site and/or the B-site are highlighted. (**c**) Matrix plot of the double perovskites bandgaps in the database^38^ used in the present study. The abscissa and ordinate represent the A–B cation pairs of the constituent single perovskites. The matrix diagonal, shown with gray circles, represents the 53 single perovskites which were not included in the database. White circles represent the 72 compositions with either zero or negligible bandgaps, which were also not included in the database. (**d**) Histogram of GLLB-SC bandgaps of 1306 double perovskites used in the development of the ML model presented here.

We start by describing the details of our double perovskite bandgap dataset that was used to train, validate and test the prediction performance of the ML models developed here. The dataset used here came from the Computational Materials Repository (CMR)^7^. The double perovskite structures reported in this dataset were obtained by combining 53 stable cubic perovskite oxides which were found to have a finite bandgap in a previous screening based on single perovskites^38^,^39^. These 53 parent single perovskites contained fourteen different A-site cations (*viz.* Ag, Ba, Ca, Cs, K, La, Li, Mg, Na, Pb, Rb, Sr, Tl and Y) and ten B-site cations (*viz.* Al, Hf, Nb, Sb, Sc, Si, Ta, Ti, V, Zr). Four cations (Ga, Ge, In and Sn) were found to appear on either A- or B-sites. The chemical space spanned by these compounds is captured in Fig. 1b.

A total of ^53^C_2_ = 1378 unique double perovskites are possible by combining the 53 stable single cubic perovskite oxides, when taken pairwise. However, out of these systems, 72 double perovskites are metallic (or have a very small bandgap 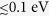) and are not included in the database. These systems are depicted in Fig. 1c as off-diagonal circles. The CMR dataset reports the electronic bandgaps of the remaining1306 unique double perovskites.

Depending on the nature of the cations, various types of cation ordering can consequently arise in the double perovskites^40^. For a doubly substituted AA’BB’O_6_-type perovskite, there are three common ways in which cations at each of the two sublattices can order, leading to a total of nine different ordered arrangements. Specifically, A and A’ (and B and B’) cations can order in layered, columnar, or rocksalt arrangements. The most commonly observed type of ordering for the B-site sublattice is the one in which the cations alternate in all three dimensions, mimicking a rocksalt-type ordered sublattice to effectively accommodate any local strain arising due to size mismatch of the two cations. Less frequently, the B-site cations may form a layered order, where they alternate only in one direction and form continuous layers in the other two normal cartesian axes. Rarely, however, a columnar order may take place, where the two different cations alternate in two orthogonal directions, but form a continuous column along the third direction. The CMR database reports the bandgaps of all the double perovskites with the rocksalt ordering of cations at both the A- and the B-sites^38^.

The reported bandgaps (*cf.* Fig. 1c,d) are computed using density functional theory (DFT)^41^ as implemented in the GPAW code^42^ with the Gritsenko, van Leeuwen, van Lenthe and Baerends potential (GLLB)^43^, further optimized for solids (-SC) by Kuisma *et al.*^44^. The GLLB functional has an inbuilt prescription for the evaluation of the derivative discontinuity^45^, which is added back to the Kohn-Sham bandgap to correct for the bandgap underestimation within conventional DFT. In fact, the GLLB-SC bandgaps for several single metal oxides have been found in excellent agreement with the corresponding experimental values (*cf.* Supplementary Information)^38^. Furthermore, the GLLB-SC functional was recently tested against the more advanced and demanding eigenvalue-self-consistent GW approach and has been shown to give good agreement for the bandgap of 20 randomly chosen systems forming an unconventional set of ternary and quaternary compounds taken from from the Materials Project database^46^. Finally, we would also like to note that despite its significantly low computational cost compared to the GW approach, the GLLB-SC functional is about twice as expensive as compared to a conventional DFT calculation employing a local or semi-local functional.

Any ML method, targeted towards learning a prespecified material property, relies on two main ingredients: the learning algorithm itself and a numerical representation (in form of a descriptor) of the materials in the learning (or training) dataset. Identification of an appropriate and most suitable fingerprint for a given prediction task is one of the central challenges, at present being actively pursued by the community. The specific choice of this numerical representation is entirely application dependent and a number of proposals in terms of high-level features (*e.g.*, *d*-band center, elemental electronegativities and ionization potentials, valence orbital radii)^26^,^47^,^48^, topological features^49^, atomic radial distribution functions^19^, compositional, configurational and motif based fingerprints^2^,^18^,^25^ have been made. Regardless of the specific choice pursued, the representations are expected to satisfy certain basic requirements such as invariance with respect to transformations of the material such as translation, rotation, and permutation of like elements. Additionally, it is also desirable that the fingerprinting be chemically intuitive and physically meaningful.

**Figure 2:**
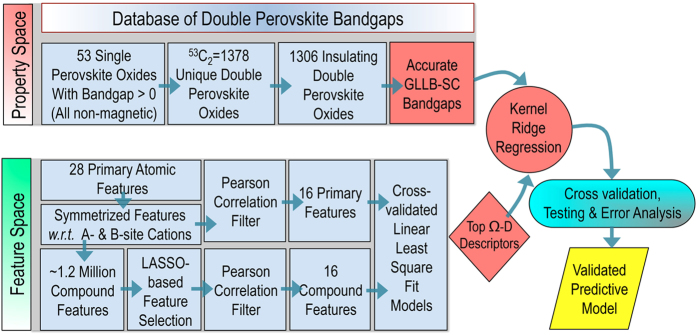
Overall workflow for the statistical learning model. Schematic presents the details of the CMR double perovskite bandgap database and outlines the workflow adopted for the primary and compound feature selection, leading to a cross-validated and tested nonlinear regression model for bandgap predictions.

The overall workflow adopted in the present study, combining an effective feature search strategy with a state-of-the-art statistical learning method, is outlined in Fig. 2. Our systematic approach starts with identification of seven atomic (or elemental) features for each of the cation species forming the double perovskite structure. These elemental features (*viz.* Pauling’s electronegativity (*χ*), ionization potential (*I*), highest occupied atomic level (*h*), lowest unoccupied atomic level (*l*), and *s*-, *p*- and *d*- valence orbital radii *r*_*s*_, *r*_*p*_ and *r*_*d*_ of isolated neutral atoms) are easily accessible and physically intuitive attributes of the constituent atoms at the A- and B-sites. While *χ*, *I*, *h* and *l* naturally form the energy scales relevant towards prediction of the bandgap, the valence orbital radii were included based on their excellent performance exhibited in classification of AB binary solids^50^,^51^. Taking these seven elemental features for each of the four atoms, occupying either A- or B-site, forms our starting set of twenty eight elemental features. Further details on the feature set are provided in the Methods section.

It is also worthwhile to note at this point that the double perovskite structure under investigation is invariant with respect to swapping of the two A-site cations as well as the two B-site cations, *i.e.*, AA’BB’O_6_, A’ABB’O_6_ and AA’B’BO_6_ are all identical systems. To incorporate this structural symmetry into the model, we symmetrize the above-mentioned 28 elemental features such that they reflect the underlying symmetry of the A- and B-site sublattices of the double perovskite crystal structure. This is achieved by taking the absolute sum 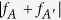 and absolute difference 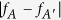 for each pair of elemental features *f*_*A*_ and *f*_*A*′_, representing the two A-site cations. Features for the B-site cations were also transformed in a similar fashion. For convenience of notation, 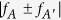 and 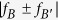 are henceforth represented by 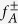 and 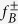, respectively. Building such symmetry at the feature level ensures that the deemed ML model would predict identical bandgaps for symmetry unique systems, irrespective of any specific labeling of the two A- and two B-site atoms. This set of 28 symmetrized features thus achieved is hereafter referred to as *primary* features.

At this point, we adopt a two-fold route for feature selection. While the primary features can directly be used in a statistical learning model, we also consider a large set of conjunctive—or compound—features built in a controlled manner to allow for non-linearity at the feature level. The compound feature set is built in the following way: 6 prototypical functions, namely, *x*, *x*^1/2^, *x*^2^, *x*^3^, ln(1 + *x*), and *e*^*x*^, with *x* being one of the 28 primary features, were considered. This immediately generates 168 features. Simply multiplying these features of single functions taken either two or three at a time leads to additional 16,464 and 1,229,312 features, respectively. This approach thus provides us with 1,245,944 compound features, each of which is a function involving up to 3 primary features. Finally, a least absolute shrinkage and selection operator (LASSO)-based model selection is employed to downselect a set of 40 compound features, which are deemed most relevant towards prediction of the bandgaps. We note here that this strategy of creating a large number of initial compound features and down-selecting to the most relevant ones using LASSO has recently been successfully employed to identify new crystal structure classifiers^51^. A LASSO-based formalism has also been employed to identify lower-dimensional representations of alloy cluster expansions^52^.

**Figure 3:**
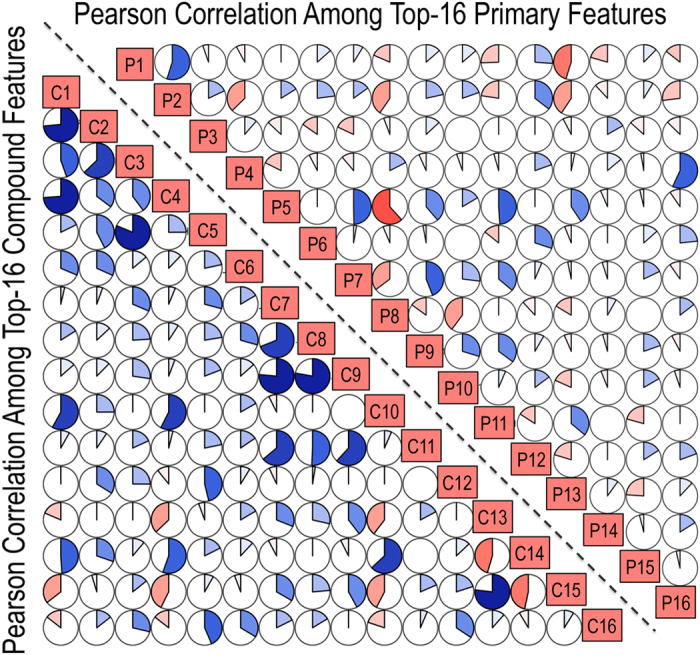
Pearson correlation map for features. A graphical representation of the Pearson correlation matrix for the downselected primary (labelled as P*i* with *i* ∈ 1,16 in the upper-right part) and compound (labelled as C*i* with *i* ∈ 1,16 in the lower-left part) features is presented. Blue and red colors indicate positive and negative correlations, respectively; the lighter the tone used, the less significant the corresponding correlation. The filled fraction of the circle in each of the pie charts corresponds to the absolute value of the associated Pearson correlation coeficient.

Next, the primary features and downselected compound features are subjected to a Pearson correlation filter (*cf.* Fig. 2) to remove features that exhibit a high correlation with the other features in each set. The cut-off of the Pearson correlation filter was adjusted such that only 16 features in each set survive. Tests showed that selecting more than 16 features does not lead to any improvements in the out-of-sample prediction accuracy of ML models. A Pearson correlation map showing the correlation for each pair of the primary or compound features is presented in Fig. 3.

The above sets of 16-primary and 16-compound features were subsequently used separately to construct all possible Ω-dimensional (or Ω−D) descriptors (*i.e.*, taking Ω features at a time), where Ω was varied from 1 to 16. This leads to 2^16^ − 1(=65535) total possible descriptors to be tested for the primary and the compound feature sets. Since testing and evaluating prediction performance of such a large number of descriptors using non-linear statistical learning models (such as kernel ridge regression or KRR) is a highly computation-time intensive task, we resort here to a cross-validated linear least square fit (LLSF) model instead. A training set consisting of 90% of the whole dataset was used to fit a linear model, and the rest 10% was used as a test set to evaluate root mean squared (*rms*) error and coefficient of determination (*R*^2^) of the fit. To take into account variability of the models, average test set *rms* error and average test set *R*^2^ over 100 different bootstrap runs were used to rank the linear models.

**Figure 4:**
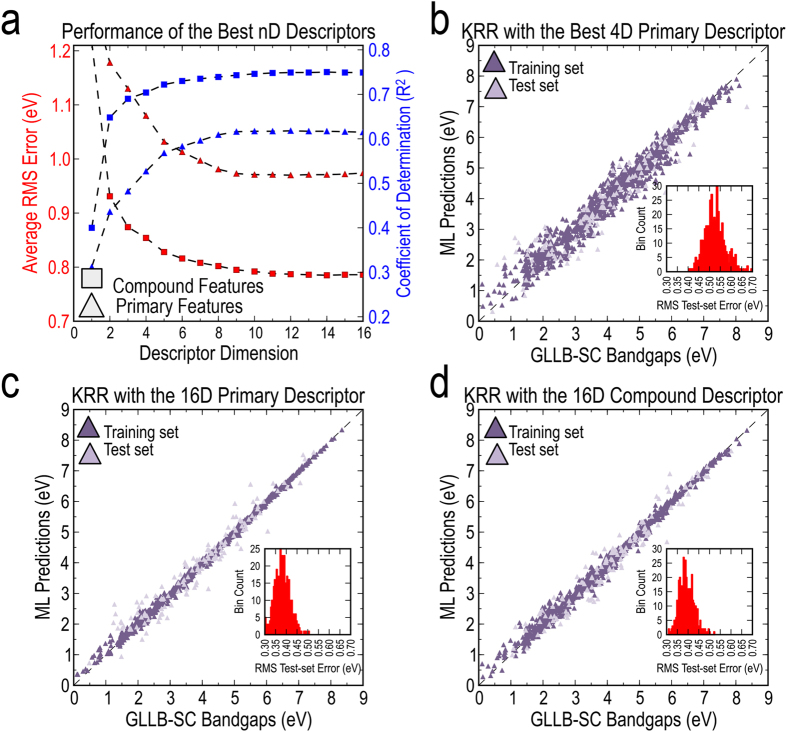
Prediction performance of the developed linear and non-linear learning models. (**a**) Computed test set *rms* errors and coefficient of determinations (*R*^2^) for the best Ω − *D* models, with Ω ranging from 1 through 16, in linear least square fit (LLSF) models which are built on either primary or compound features. The prediction performance reported here is computed as an average over 100 different runs, each with randomly selected 90% training and 10% test sets. Representative parity plots comparing the DFT-computed bandgaps against the KRR predicted bandgaps, for (**b**) the 4-D primary descriptor, (**c**) the 16-D primary descriptor and (**d**) the 16-D compound descriptor. Histograms of the test set average *rms* error, computed over 400 different runs, are also presented as insets in the last three panels.

The LLSF performance of the best Ω−D descriptors for a given Ω ∈[1, 16] is presented in Fig. 4a. We find that for any given Ω, the descriptors with the compound features perform much better than those formed using the primary features. Certainly, this boost in performance can be attributed to the additional flexibility imparted by the non-linear functions in the compound features. Furthermore, a compound feature can effectively have a combination of up to three functions of primary features. We also note that going beyond a 10-D descriptor does not improve the prediction performance in either case (*cf.* Fig. 4a). For instance, the average *rms* error for the 16-D descriptor formed with compound features is 0.786 eV, while that for the 10-D descriptor is 0.792 eV. The average *rms* errors for the corresponding descriptors with primary features are 0.971 eV and 0.973 eV, respectively.

Performance of the best primary and compound Ω − D descriptors (with Ω ∈[1, 16]) identified above was then reassessed in a Kernel ridge regression (KRR)^53^,^54^ model—a state-of-the-art ML method capable of handling complex non-linear relationships—which has recently been shown to be promising for prediction of a diverse set of materials properties^2^,^17^,^55^,^56^,^57^. Based on the principle of similarity, the KRR method first uses a distance measure such as the Euclidean norm in the descriptor space (*i.e.*, 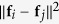, for *i*^*th*^ and *j*^*th*^ compounds in the training set) to quantify (dis)similarity between materials; the property to be predicted is then computed as a linear combination of the kernel (*e.g.,* a gaussian kernel 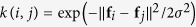 used in the present case) distance functions of materials of interest and the training set materials. Therefore, constructing descriptors in which materials have a small distance when their property of interest is similar is of particular importance for the learning process. Further details on the KRR learning model are provided in Methods.

Results obtained using the cross-validated KRR models are presented in Table 1 and the identities of the best Ω−D descriptors are provided in the Supplementary Information. For each descriptor, the average *rms* error and average *R*^2^ on training and test sets are reported. The average was taken over 100 different KRR runs, in each of which a 90% training set and a 10% test set were randomly selected. Not surprisingly, it is seen that both the learning and prediction accuracies grow with the descriptor dimensionality (and complexity). Interestingly, unlike the LLSF model, in the KRR model the performance of a primary descriptor is found to be comparable to that of the corresponding compound descriptor. This is owing to the inclusion of the nonlinearity in the learning model itself, which boosts the performance of the primary descriptors. In light of this observation, going forward with the KRR model, we choose the simpler models with the primary descriptors over the compound descriptors.

It is interesting to note that going from the 3-D to the 4-D primary descriptor (*cf.* Table 1) leads to a significant improvement in the model prediction performance. For instance, the average *R*^2^ on the test set increases from 0.69 to 0.90 and average *rms* error decreases from ~0.87 eV to ~0.50 eV. Going beyond the 4-D descriptor, however, only results in marginal improvements. For instance, with the 16-D descriptor (containing all of the primary features) the obtained average test set *R*^2^ is ~0.94, only slightly better than that of the 4-D descriptor. Figure 4b–d compare the KRR prediction performance of the 4-D descriptor with the 16-D primary and the 16-D compound descriptor in separate parity plots, using a representative training/test set split. It can be seen that while the training set performance is significantly better in the KRR models with the higher dimensional descriptors, the test set performance of those models can be considered comparable (or only slightly better) to that of the 4-D descriptor.

## Discussion

We now examine the individual primary features that combine to give the 4-D primary descriptor. These features are: 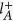, 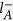, 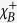, 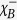, *i.e.*, the absolute sum and difference of elemental lowest occupied levels of the two A-site atoms and the electronegativities of the two B site atoms. We also note that the two conjugate pairs of these elemental features appear together and none of the primary features with valence orbital radii appears. Furthermore, the descriptor is well balanced with respect to the participation from the features specific to the A-site atoms (*i.e.*, 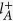, 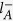) and to the B-site atoms (*i.e.*, 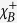, 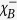). In addition to being chemically intuitive, elegant and symmetric, the identified descriptor is also simple and easily accessible. It is always desirable to have a ML prediction model built on simpler (*i.e.*, low dimensional) and intuitive descriptors, since with high dimensional complex descriptors there is always a danger of overfitting leading to poor model generalizability. Therefore, by preferring the 4-D primary descriptor over the 16-D descriptor, we are trading some model accuracy for model simplicity and better model generalizability.

**Figure 5:**
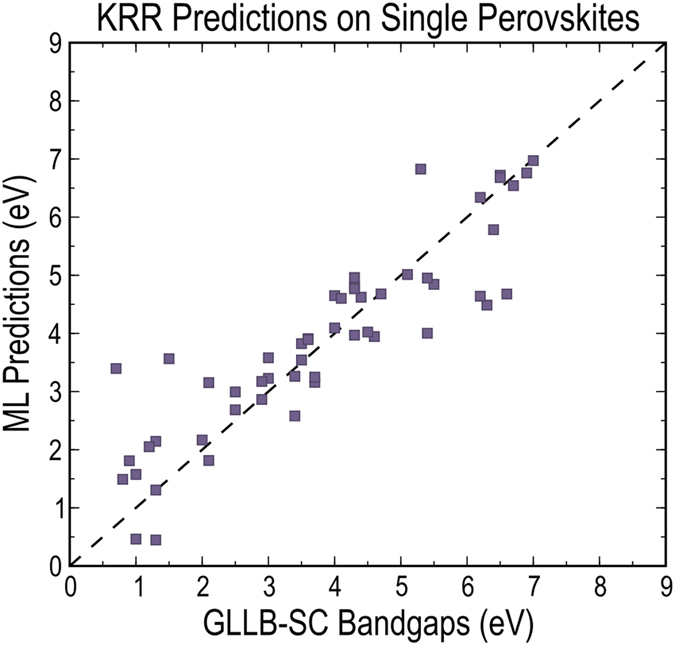
Predictions on single perovskites. Prediction performance of the KRR model in predicting the bandgaps of the parent 53 single perovskites, which form all the double perovskites in the database. The model was trained on a randomly selected 90% training set from the double perovskite bandgap database using the 4-D primary descriptor.

To further test the model’s predictability, we used the cross-validated KRR learning model, trained on a randomly selected 90% double perovskite dataset, to predict bandgaps of the original 53 parent single perovskites. We note that for the single perovskites, owing to the constraints A = A’ and B = B’, only two of the four features survive (i.e., for all the single perovskites we have 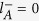, 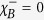). Figure 5 compares the bandgaps predicted by the model with those computed using DFT with the GLLB-SC functional. Given that the model was never trained on single perovskites and that only two of the four primary features effectively survive for a single perovskite, such a prediction performance is rather remarkable.

**Figure 6:**
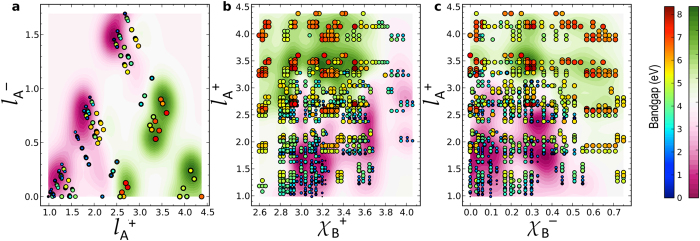
Feature-property maps. (**a**–**c**) Two dimensional contour maps showing regions of relatively larger and smaller bandgaps in the feature space. In each of the panels, only two of the four features are explicitly presented, while the dependency of the other two features has been integrated out (see text for details). The data-points in the double perovskites dataset are also plotted on top of the contour plots. The marker size and color of the scattered points represent their bandgaps.

To gain deeper insight into the model’s remarkable prediction performance, we next construct 2-D contour plots in which dependence of any two of the four features has been marginalized (by considering an averaged value along those particular dimensions, as explained below). We start with a fine 4-D grid in the feature space constituted by the four primary features identified above, while still confining ourselves within the boundaries of the original feature space used to train the KRR model. Each point on this grid then, in principle, represents a descriptor. Next, we use the trained KRR model to make predictions using each of these descriptor points as a model input. For the sake of better representation, we convert the predictions in this 4-D feature space into a 2-D plot by averaging out any given two of the four primary features. This approach allows us to explicitly visualize the dependence of the bandgap along any two pre-specified features, while the dependence of other two features is considered only in an *averaged* manner. We can now represent this data in a 2-D contour plot. Three out of a total of six such possible plots are shown in Fig. 6, where green and purple regions represent the high- and low-bandgap regions, respectively. The data-points in the double perovskites dataset are also plotted on top for validation, color (or size) coded according to their GLLB-SC bandgaps. Since the dependence of two out of the four features has already been integrated out, one does not expect a quantitative agreement between the contour and scatter plots. However, it can be seen from the figure that the two are in quite good agreement. The green “mountains” on the contour plot are largely occupied by red (large) circles while the purple “valleys” are mostly populated with blue (small) circles. Such feature-property maps provide a pathway towards drawing decision rules (for a targeted functionality) from statistical learning models. Furthermore, while the original model can be used to make quantitative predictions, such simple feature-property maps can be employed as a first-line of screening to make qualitative predictions or devise simple screening criteria for a given property (in our case the bandgap).

Finally, we comment about the limitations and domain of applicability of the ML model. The presented model is applicable within the considered chemical space (*i.e.*, aforementioned choices of A- and B-site cations) and to non-magnetic AA’BB’O_6_ type perovskites, which can be separated into two charge neutral ABO_3_ and A’B’O_3_ single perovskites. Test performance on double perovskite compounds which cannot be decomposed in such a manner was found to be poor, which is not surprising since the learning model was never trained on such compounds. Extending the ML model to such compounds and accounting for other possible A- and B-site cation orderings remains work to be undertaken in future studies. It will also be interesting to check the general applicability of the identified descriptor by employing it to predict the bandgaps of other materials classes, quite distinct from perovskites and related chemistries.

In summary, we have presented a robust ML model along with a simple elemental descriptor set for efficient predictions of electronic bandgaps of double perovskites. The proposed optimal descriptor set was identified via searching a large part of feature space that involved more than ~1.2 million descriptors formed by combining simple elemental features such as electronegativities, ionization potentials, electronic energy levels and valence orbital radii of the constituent atomic species. The KRR-based statistical learning model developed here was trained and tested on a database consisting of accurate bandgaps of ~1300 double perovskites computed using the GLLB-SC functional within the framework of density functional theory. One of the most important chemical insights that came out of the adopted learning framework is that the bandgap is primarily controlled (and therefore can efficiently be learned) by the lowest occupied energy levels of the A-site elements and electronegativities of the B-site elements. The average test set *rms* error of the cross validated model with only four primary features (i.e., the 4-D primary descriptor) is found to be 0.5 eV, which is further reduced to ~0.37 eV (0.36 eV) with the primary (compound) 16-D descriptor. Out-of-sample prediction performance of the trained model is further demonstrated by its ability to predict bandgaps of several single perovskites. Finally we have shown that the prediction performance of the model can be visually rationalized by constructing several two-dimensional feature-property contour maps. We believe that the ML approach presented here is general and can be applied to any material class in a restricted chemical space with a given crystal structure to make efficient predictions of bandgaps. Such a prediction strategy can be practically useful in an initial screening to identify promising candidates in a high throughput manner.

## Methods

### Details of feature set

For feature set accumulation, we start from 7 atomic features for each metal atom A in the double perovskite structure. These primary atomic features are Pauling’s electronegativity (*χ*), ionization potential (*I*), highest occupied atomic Kohn-Sham level (*h*), lowest unoccupied atomic Kohn-Sham level (*l*), and *s*-, *p*- and *d*- Zunger’s valence orbital radii *r*_*s*_, *r*_*p*_ and *r*_*d*_ of isolated neutral atoms^50^. The ionization potential and Pauling’s electronegativity data were taken from the literature^6^,^59^ and the highest-occupied lowest-unoccupied Kohn-Sham levels of the isolated atomic species were computed using the GGA-PBE exchange-correlation functional^58^.

### Machine learning model

Within the present similarity-based KRR learning model, the bandgap of a system in the test set is given by a sum of weighted Gaussians over the entire training set. As a part of the model training process, the learning is performed by minimizing the expression 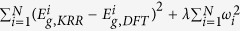, with 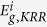 being the KRR estimated bandgap value, 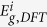 the DFT value, and *λ* a regularization parameter. The explicit solution to this minimization problem is 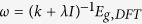, where *I* is the identity matrix, and 
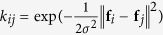
 is the kernel matrix elements of all compounds in the training set. The parameters *λ*, *σ* are determined in an inner loop of fivefold cross validation using a logarithmically scaled fine grid. We note that KRR training and hyperparameter determination were performed only using the training data and the test set samples were never seen by the KRR model during the training procedure.

## Additional Information

**How to cite this article**: Pilania, G. *et al.* Machine learning bandgaps of double perovskites. *Sci. Rep.* **6**, 19375; doi: 10.1038/srep19375 (2016).

## Supplementary Material

Supplementary Information

## Figures and Tables

**Table 1:** Prediction performance of the KRR models.

Performance of KRR ML models with primary descriptors					Performance of KRR ML models with compound descriptors				
Descriptor	Training set (90%)		Test set (10%)		Descriptor	Training set (90%)		Test set (10%)	
Dimension	*rms* error (eV)	*R*^2^	*rms* error (eV)	*R*^2^	Dimension	*rms* error (eV)	*R*^2^	*rms* error (eV)	*R*^2^
1-D	0.959	0.634	1.056	0.546	1-D	0.954	0.638	1.056	0.545
2-D	1.059	0.554	1.114	0.493	2-D	0.888	0.686	0.879	0.685
3-D	0.689	0.811	0.867	0.692	3-D	0.774	0.762	0.777	0.753
4-D	0.306	0.963	0.501	0.897	4-D	0.716	0.796	0.742	0.775
5-D	0.270	0.971	0.529	0.885	5-D	0.566	0.872	0.637	0.834
6-D	0.302	0.968	0.521	0.889	6-D	0.502	0.900	0.594	0.855
7-D	0.356	0.949	0.510	0.892	7-D	0.426	0.928	0.554	0.875
8-D	0.281	0.969	0.429	0.925	8-D	0.437	0.924	0.563	0.870
9-D	0.219	0.981	0.406	0.932	9-D	0.399	0.937	0.550	0.876
10-D	0.139	0.992	0.397	0.935	10-D	0.300	0.964	0.484	0.904
11-D	0.178	0.987	0.413	0.930	11-D	0.249	0.975	0.455	0.915
12-D	0.074	0.998	0.393	0.936	12-D	0.222	0.980	0.451	0.917
13-D	0.137	0.993	0.365	0.944	13-D	0.187	0.986	0.418	0.928
14-D	0.110	0.995	0.397	0.935	14-D	0.169	0.989	0.413	0.930
15-D	0.087	0.997	0.377	0.941	15-D	0.144	0.992	0.376	0.942
16-D	0.080	0.997	0.371	0.939	16-D	0.132	0.993	0.360	0.947

Computed training set and test set *rms* errors and coefficient of determinations (*R*^2^) for the best Ω − *D* models, with Ω ∈[1, 16], in 5-fold cross-validated KRR models built on top primary and compound descriptors (identified in the LLSF models). The reported *rms* errors and *R*^2^ are averaged values over 100 different runs, each with randomly selected 90% training and 10% test sets. Here 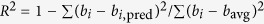 with *b*_*i*_, *b*_*i*,pred_ and *b*_avg_ being the GLLB-SC computed bandgaps, model-predicted bandgaps and average bandgap value in the training or test set.

## References

[b1] Curtarolo S. *et al.* The high-throughput highway to computational materials design. Nat. Mater. 12, 191–201 (2013). 23422720 10.1038/nmat3568

[b2] Pilania G., Wang C., Jiang X., Rajasekaran S. & Ramprasad R. Accelerating materials property predictions using machine learning. Sci. rep. 3, 2810 (2013). 24077117 10.1038/srep02810 PMC3786293

[b3] Sharma V. *et al.* Rational design of all organic polymer dielectrics. Nat. comm. 5, 4845 (2014). 10.1038/ncomms5845 25229753

[b4] Ceder G., Hauthier G., Jain A. & Ong S. P. Recharging lithium battery research with first-principles methods. Mater. Res. Soc. Bull. 36, 185–191 (2011).

[b5] Curtarolo S. *et al.* AFLOWLIB.ORG: AFLOWLIB. ORG: A distributed materials properties repository from high-throughput ab initio calculations. Comput. Mater. Sci. 58, 227 (2012).

[b6] *Materials Project - A Materials Genome Approach*, http://materialsproject.org/ (accessed: 15th October 2015).

[b7] *Computational Materials Repository* https://wiki.fysik.dtu.dk/cmr/ (Documentation) and https://cmr.fysik.dtu.dk/ (accessed: 15th October 2015).

[b8] Service R. F. Materials scientists look to a data-intensive future. Science 335, 1434–1435 (2012). 22442457 10.1126/science.335.6075.1434

[b9] Flach P. Machine Learning: The Art and Science of Algorithms that Make Sense of Data (Cambridge University Press, Cambridge, 2012).

[b10] Burbidge R., Trotter M., Buxton B. & Holden S. Drug design by machine learning: support vector machines for pharmaceutical data analysis. Computers & chemistry 26, 5–14 (2001). 11765851 10.1016/s0097-8485(01)00094-8

[b11] Jones N. *Quiz-playing computer system could revolutionize research.* Nature News (2011), Available at: http://dx.doi.org/10.1038/news.2011.95. (Accessed: 23rd November 2015).

[b12] MacLeod N., Benfield M. & Culverhouse P. Time to automate identification. Nature 467, 154–155 (2010). 20829777 10.1038/467154a

[b13] Abu-Mostafa Y. S. Machines that Think for Themselves. Sci Am 307, 78–81 (2012). 22779276

[b14] Silver N. The Signal and the Noise: Why So Many Predictions Fail but Some Don’t (Penguin Press, New York, 2012).

[b15] Mueller T., Kusne A. G. & Ramprasad R. Machine learning in materials science: Recent progress and emerging applications. Rev. Comput. Chem. (Accepted for publication).

[b16] Rajan K. in Informatics for Materials Science and Engineering: Data-driven Discovery for Accelerated Experimentation and Application (ed. Rajan K.), Ch. 1, 1–16 (Butterworth-Heinemann, Oxford, 2013).

[b17] Rupp M., Tkatchenko A., Muller K.-R. & von Lilienfeld O. A. Fast and accurate modeling of molecular atomization energies with machine learning. Phys. Rev. Lett. 108, 058301 (2012). 22400967 10.1103/PhysRevLett.108.058301

[b18] Huan T. D., Mannodi-Kanakkithodi A. & Ramprasad R. Accelerated materials property predictions and design using motif-based fingerprints, Phys. Rev. B 92, 014106 (2015).

[b19] Schütt K. T. *et al.* How to represent crystal structures for machine learning: Towards fast prediction of electronic properties. Phys. Rev. B 89, 205118 (2014).

[b20] Meredig B. *et al.* Combinatorial screening for new materials in unconstrained composition space with machine learning. Phys. Rev. B 89 094104 (2014).

[b21] Faber F., Lindmaa A., von Lilienfeld O. A. & Armiento R. Crystal Structure Representations for Machine Learning Models of Formation Energies. Int. J. Quantum. Chem. 115, 1094–1101 (2015).

[b22] Faber F., Lindmaa A., von Lilienfeld O. A. & Armiento R. Machine Learning Energies of 2 M Elpasolite (ABC2D6) Crystals. http://arxiv.org/abs/1508.05315 (2015). 10.1103/PhysRevLett.117.135502 27715098

[b23] Pozun Z. *et al.* Optimizing transition states via kernel-based machine learning. Chem. Phys. 136, 174101 (2012). 10.1063/1.4707167 22583204

[b24] Behler J. Atom-centered symmetry functions for constructing high-dimensional neural network potentials. J. Chem. Phys. 134, 074106 (2011). 21341827 10.1063/1.3553717

[b25] Botu V. & Ramprasad R. Adaptive machine learning framework to accelerate ab initio molecular dynamics, Int. J. Quantum Chem. 115, 1074–1083 (2015).

[b26] Pilania G., Gubernatis J. E. & Lookman T. Structure classification and melting temperature prediction in octet AB solids via machine learning. Phys. Rev. B 91, 214302 (2015).

[b27] Pilania G., Gubernatis J. E. & Lookman T. Classification of octet AB-type binary compounds using dynamical charges: A materials informatics perspective. accepted for publication in Sci. Rep. (2015). 10.1038/srep17504 PMC4668360 26631979

[b28] Pilania G., Balachandran P. V., Gubernatis J. E. & Lookman T. Predicting the formability of ABO_3_ perovskite solids: A machine learning study. Acta Cryst. B 71, 507–513 (2015). 10.1107/S2052520615013979 26428400

[b29] Snyder J. C., Rupp M., Hansen K., Müller K. R. & Burke K. Finding density functionals with machine learning. Phys. Rev. Lett. 108, 253002 (2012). 23004593 10.1103/PhysRevLett.108.253002

[b30] Lee J., Seko A., Shitara K. & Tanaka I. Prediction model of band-gap for AX binary compounds by combination of density functional theory calculations and machine learning techniques. arXiv preprint arXiv:1509.00973 (2015).

[b31] Dey P. *et al.* Informatics-aided bandgap engineering for solar materials. Com. Mat. Sci. 83, 185–195 (2014).

[b32] Mitchell R. H. Perovskites: Modern and Ancient (Almaz Press, Ontario, Canada, 2002).

[b33] Setyawan W., Gaume R. M., Lam S., Feigelson R. S. & Curtarolo S. High-throughput combinatorial database of electronic band structures for inorganic scintillator materials. ACS Comb. Sci. 13, 382–390 (2011). 21644557 10.1021/co200012w

[b34] Olivares-Amaya R. *et al.* Accelerated computational discovery of high-performance materials for organic photovoltaics by means of cheminformatics. Energy Environ. Sci. 4, 4849 (2011).

[b35] Chemical Bonding at Surfaces and Interfaces (Eds Nilsson A., Pettersson L. G. M. & Nørskov J. K.) (Elsevier, Amsterdam, The Netherlands, 2008).

[b36] Hedin L. New method for calculating the one-particle Green’s function with application to the electron-gas problem. Phys. Rev. 139 A796 (1965).

[b37] Heyd J., Scuseria G. E. & Ernzerhof M. Hybrid functionals based on a screened Coulomb potential. J. Chem. Phys. 124, 219906 (2006).

[b38] Castelli I. E. *et al.* Computational screening of perovskite metal oxides for optimal solar light capture. Energy Environ. Sci. 5, 5814 (2012).

[b39] Castelli I. E., Thygesen K. S. & Jacobsen K. W. Bandgap engineering of double perovskites for one-and two-photon water splitting. MRS Proceedings 1523, mrsf12-1523-qq07-06 (2013), 10.1557/opl.2013.450.

[b40] Vasala S. & Karppinen M. A_2_B’B”O_6_ perovskites: A review. Prog. Solid State Chem. 43, 1–36 (2015).

[b41] Martin R. Electronic Structure: Basic Theory and Practical Methods (Cambridge University Press, New York, 2004).

[b42] Mortensen J. J., Hansen L. B. & Jacobsen K. W. Real-space grid implementation of the projector augmented wave method. Phys. Rev. B 71, 35109 (2005).

[b43] Gritsenko O., van Leeuwen R., van Lenthe E. & Baerends E. J. Self-consistent approximation to the Kohn-Sham exchange potential. Phys. Rev. A 51, 1944 (1995). 9911804 10.1103/physreva.51.1944

[b44] Kuisma M., Ojanen J., Enkovaara J. & Rantala T. T. Kohn-Sham potential with discontinuity for band gap materials. Phys. Rev. B 82, 115106 (2010).

[b45] Talman J. D. & Shadwick W. F. Optimized effective atomic central potential. Phys. Rev. A 14, 36 (1976).

[b46] Castelli I. E. *et al.* New light-harvesting materials using accurate and efficient bandgap calculations. Adv. Energy Mater. 5, 1400915 (2015).

[b47] Andriotis A. N. *et al.* Informatics guided discovery of surface structure-chemistry relationships in catalytic nanoparticles. J. Chem. Phys. 140, 094705 (2014). 24606374 10.1063/1.4867010

[b48] Dam H. C., Pham T. L., Ho T. B., Nguyen A. T. & Nguyen V. C. Data mining for materials design: A computational study of single molecule magnet. J. Chem. Phys. 140, 044101 (2014). 25669499 10.1063/1.4862156

[b49] Brown R. D. & Martin Y. C. The information content of 2D and 3D structural descriptors relevant to ligand-receptor binding. J. Chem. Inf. Comput. Sci. 37, 1 (1997).

[b50] Zunger A. Systematization of the stable crystal structure of all AB-type binary compounds. Phys. Rev. B 22, 5839 (1980).

[b51] Ghiringhelli L. M., Vybiral J., Levchenko S. V., Draxl C. & Scheffler M. Big data of materials science: Critical role of the descriptor. Phys. Rev. Lett. 114, 105503 (2015). 25815947 10.1103/PhysRevLett.114.105503

[b52] Nelson L. J., Hart G. L., Zhou F. & Ozoliņš V. Compressive sensing as a paradigm for building physics models. Phys. Rev. B 87, 035125 (2013).

[b53] Hastie T., Tibshirani R. & Friedman J. The Elements of Statistical Learning: Data Mining, Inference, and Prediction (Springer, New York, 2009).

[b54] Müller K.-R., Mika S., Ratsch G., Tsuda K. & Scholkopf B. An introduction to kernel-based learning algorithms. IEEE Trans Neural Netw 12, 181–201 (2001). 18244377 10.1109/72.914517

[b55] Bereau T., Andrienko D. & von Lilienfeld O. A. Transferable atomic multipole machine learning models for small organic molecules. J. Chem. Theory Comput. 11, 3225–3233 (2015). 26575759 10.1021/acs.jctc.5b00301

[b56] Hansen K. *et al.* Assessment and validation of machine learning methods for predicting molecular atomization energies. J. Chem. Theory Comput. 9, 3404 (2013). 26584096 10.1021/ct400195d

[b57] Lopez-Bezanilla A. & von Lilienfeld O. A. Modeling electronic quantum transport with machine learning. Phys. Rev. B 89, 235411 (2014).

[b58] Perdew J. P., Burke K. & Ernzerhof M. Generalized gradient approximation made simple. Phys. Rev. Lett. 77, 3865 (1996). 10062328 10.1103/PhysRevLett.77.3865

[b59] Lide D. R. Handbook of Chemistry and Physics (CRC Press, Boston, 2004).

