# Supplementary material for: Machine learning bandgaps of double perovskites
Source: Sci Rep. 2016 Jan 19;6:19375. doi: 10.1038/srep19375 (PMC4726030; doi:10.1038/srep19375)
Supplement: Supplementary Information [file srep19375-s1.pdf]

Supplemental Information for
Machine learning bandgaps of double perovskites

G. Pilania[1,*], A. Mannodi-Kanakkithodi[2], B. P. Uberuaga[1], R. Ramprasad[2], J. E. Gubernatis[3], and T. Lookman[3]

[1]Materials Science and Technology Division, Los Alamos National Laboratory, Los Alamos 87545, NM, USA
[2]Department of Materials Science & Engineering and Institute of Materials Science, University of Connecticut, Storrs, 06269 CT USA
[3]Theoretical Division, Los Alamos National Laboratory, Los Alamos 87545, NM, USA
[*]gpilania@lanl.gov

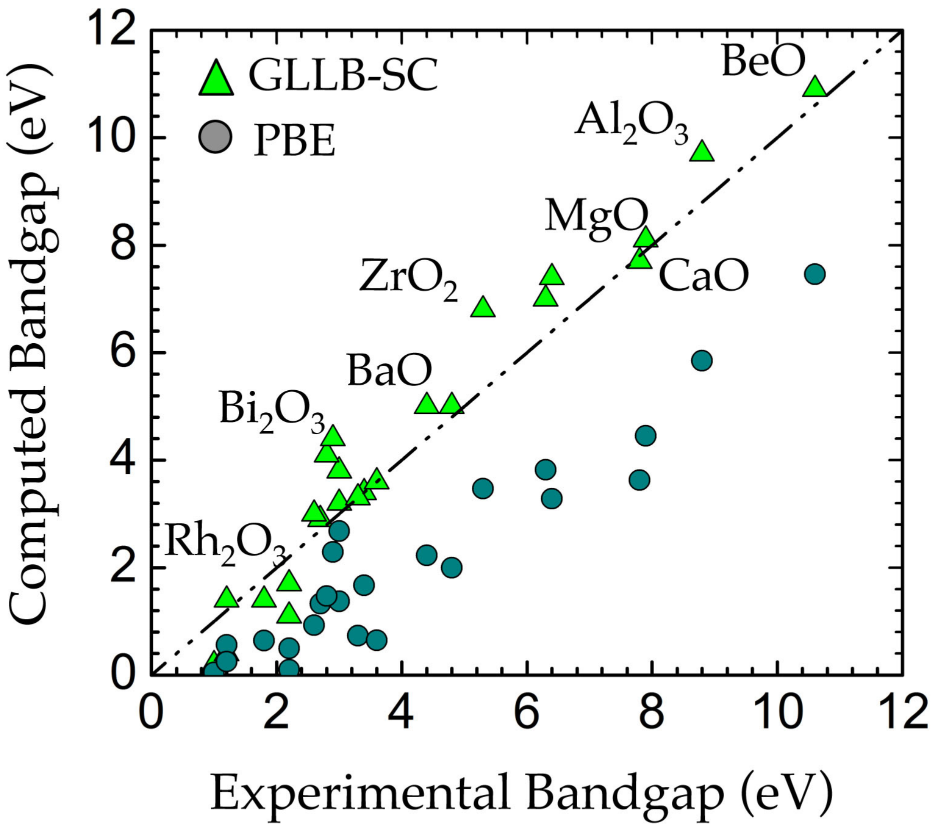

Figure 1. Parity plot comparing the PBE and GLLB-SC bandgaps with the corresponding experimentally measured bandgaps for selected single metal oxides. The plotted data is from Ref. [1].

Table 1. **Identified optimal primary Ω-D descriptors**.

**Primary features and optimal primary Ω-D descriptors**			
Down-selected primary features		Identities of the primary descriptors	
Primary feature	Feature representation	Descriptor dimension	Descriptor representation
f_1	r_{dA}^{+}	1-D	$[f_2]$
f_2	l_A^{+}	2-D	$[f_2, f_{13}]$
f_3	h_A^{+}	3-D	$[f_2, f_9, f_{13}]$
f_4	χ_A^{-}	4-D	$[f_2, f_5, f_9, f_{12}]$
f_5	χ_B^{+}	5-D	$[f_2, f_5, f_9, f_{12}, f_{13}]$
f_6	r_{dA}^{-}	6-D	$[f_2, f_3, f_5, f_9, f_{12}, f_{13}]$
f_7	I_B^{-}	7-D	$[f_2, f_3, f_5, f_7, f_9, f_{12}, f_{13}]$
f_8	r_{dB}^{+}	8-D	$[f_2, f_3, f_5, f_7, f_9, f_{12}, f_{13}, f_{15}]$
f_9	χ_B^{-}	9-D	$[f_2, f_3, f_4, f_5, f_7, f_9, f_{12}, f_{13}, f_{15}]$
f_{10}	r_{dB}^{-}	10-D	$[f_2, f_3, f_4, f_5, f_7, f_9, f_{12}, f_{13}, f_{15}, f_{16}]$
f_{11}	I_B^{+}	11-D	$[f_1, f_2, f_3, f_4, f_5, f_7, f_9, f_{12}, f_{13}, f_{15}, f_{16}]$
f_{12}	l_A^{-}	12-D	$[f_1, f_2, f_3, f_4, f_5, f_7, f_9, f_{12}, f_{13}, f_{14}, f_{15}, f_{16}]$
f_{13}	h_B^{+}	13-D	$[f_1, f_2, f_3, f_4, f_5, f_7, f_9, f_{11}, f_{12}, f_{13}, f_{14}, f_{15}, f_{16}]$
f_{14}	h_A^{-}	14-D	$[f_1, f_2, f_3, f_4, f_5, f_6, f_7, f_9, f_{11}, f_{12}, f_{13}, f_{14}, f_{15}, f_{16}]$
f_{15}	h_B^{-}	15-D	$[f_1, f_2, f_3, f_4, f_5, f_6, f_7, f_9, f_{10}, f_{11}, f_{12}, f_{13}, f_{14}, f_{15}, f_{16}]$
f_{16}	r_{sA}^{-}	16-D	$[f_1, f_2, f_3, f_4, f_5, f_6, f_7, f_8, f_9, f_{10}, f_{11}, f_{12}, f_{13}, f_{14}, f_{15}, f_{16}]$

Table 2. Identified optimal compound Ω-D descriptors.

**Compound features and optimal compound Ω-D descriptors**			
Down-selected compound features		Identities of the compound descriptors	
Compound feature	Feature representation	Descriptor dimension	Descriptor representation
f_1	$(r_{sA}^+)^2 \cdot (I_A^+)^3 \cdot \sqrt{(l_A^+)}$	1-D	$[f_1]$
f_2	$(r_{sA}^+)^2 \cdot (I_A^+)^3 \cdot exp(\chi_B^-)$	2-D	$[f_2, f_{15}]$
f_3	$(h_A^+)^2 \cdot (r_{pB}^+)^3 \cdot exp(l_B^-)$	3-D	$[f_2, f_3, f_{15}]$
f_4	$(I_A^+)^3 \cdot (l_A^+)^3 \cdot exp(r_{pB}^+)$	4-D	$[f_2, f_3, f_{11}, f_{15}]$
f_5	$(r_{pB}^+)^3 \cdot \sqrt{(h_A^+)} \cdot exp(l_B^-)$	5-D	$[f_1, f_3, f_9, f_{12}, f_{15}]$
f_6	$(r_{dA}^+)^3 \cdot exp(r_{pB}^-) \cdot exp(I_A^-)$	6-D	$[f_1, f_3, f_6, f_8, f_{12}, f_{15}]$
f_7	$(\chi_A^+)^3 \cdot exp(r_{dB}^+) \cdot exp(l_A^-)$	7-D	$[f_1, f_2, f_3, f_6, f_8, f_{12}, f_{15}]$
f_8	$\sqrt{(h_A^-)} \cdot ln(1+\chi_A^-) \cdot ln(1+l_A^-)$	8-D	$[f_1, f_2, f_3, f_6, f_8, f_{11}, f_{12}, f_{15}]$
f_9	$(h_B^+)^2 \cdot (\chi_A^+)^3 \cdot ln(1+l_A^-)$	9-D	$[f_1, f_2, f_3, f_6, f_8, f_{10}, f_{11}, f_{12}, f_{15}]$
f_{10}	$(l_A^+)^3 \cdot (l_A^+)^3 \cdot ln(1+l_A^-)$	10-D	$[f_1, f_2, f_3, f_6, f_8, f_{11}, f_{12}, f_{13}, f_{15}, f_{16}]$
f_{11}	$(\chi_A^+)^3 \cdot \sqrt{(l_A^-)} \cdot exp(h_B^-)$	11-D	$[f_1, f_2, f_3, f_6, f_8, f_{11}, f_{12}, f_{13}, f_{14}, f_{15}, f_{16}]$
f_{12}	$(r_{dB}^+)^3 \cdot (h_B^+)^3 \cdot (l_B^-)^3$	12-D	$[f_1, f_2, f_3, f_6, f_8, f_{10}, f_{11}, f_{12}, f_{13}, f_{14}, f_{15}, f_{16}]$
f_{13}	$\sqrt{(\chi_A^+)} \cdot \sqrt{(h_B^+)} \cdot \sqrt{(r_{sA}^+)}$	13-D	$[f_1, f_2, f_3, f_6, f_7, f_8, f_{10}, f_{11}, f_{12}, f_{13}, f_{14}, f_{15}, f_{16}]$
f_{14}	$(l_A^+)^2 \cdot (r_{pB}^+)^3 \cdot exp(I_B^-)$	14-D	$[f_1, f_2, f_3, f_6, f_7, f_8, f_9\ f_{10}, f_{11}, f_{12}, f_{13}, f_{14}, f_{15}, f_{16}]$
f_{15}	$(\chi_B^+)^2 \cdot \sqrt{(\chi_A^+)} \cdot \sqrt{(L_B^+)}$	15-D	$[f_1, f_2, f_3, f_5, f_6, f_7, f_8, f_9\ f_{10}, f_{11}, f_{12}, f_{13}, f_{14}, f_{15}, f_{16}]$
f_{16}	$(r_{dB}^+)^2 \cdot \sqrt{(I_A^-)} \cdot exp(L_B^-)$	16-D	$[f_1, f_2, f_3, f_4, f_5, f_6, f_7, f_8, f_9, f_{10}, f_{11}, f_{12}, f_{13}, f_{14}, f_{15}, f_{16}]$

References

1. Castelli, I.E., Olsen, T., Datta, S., Landis, D.D., Dahl, S., Thygesen, K.S., Jacobsen, K.W. Energy Environ. Sci. 5, 5814 (2012).
